# Association between combinations of genetic polymorphisms and epidemiopathogenic forms of bovine paratuberculosis

**DOI:** 10.1016/j.heliyon.2018.e00535

**Published:** 2018-03-01

**Authors:** Ramon A. Juste, Patricia Vazquez, Otsanda Ruiz-Larrañaga, Mikel Iriondo, Carmen Manzano, Mikel Agirre, Andone Estonba, Maria V. Geijo, Elena Molina, Iker A. Sevilla, Marta Alonso-Hearn, Nieves Gomez, Valentin Perez, Adoracion Cortes, Joseba M. Garrido

**Affiliations:** a Animal Health Department, NEIKER-Tecnalia, Berreaga, 1, 48160 Derio, Bizkaia, Spain; b SERIDA, Regional Service of Agricultural Research and Development, Ctra. Oviedo s/n, 33300 Villaviciosa, Asturias, Spain; c SALUVET, Animal Health Department, Faculty of Veterinary Sciences, Complutense University of Madrid, Ciudad Universitaria s/n, 28040 Madrid, Spain; d Department of Genetics, Physical Anthropology and Animal Physiology, Faculty of Science and Technology, University of the Basque Country (UPV/EHU), 48940 Leioa, Bizkaia, Spain; e Animal Health Department, Faculty of Veterinary Sciences, University of León, Campus de Vegazana s/n, 24071 León, Spain; f Department of Health, Basque Government, Matadero Frigorífico Donostiarra, S.A.L. (MAFRIDO), P° Ubarburu 16, 20014 Donostia-San Sebastian, Spain

**Keywords:** Infectious disease, Veterinary medicine, Immunology, Genetics

## Abstract

Control of major mycobacterial diseases affecting livestock is a challenging issue that requires different approaches. The use of genetic markers for improving resistance to *Mycobacterium avium* subsp. *paratuberculosis* infection in cattle has been explored as a promising population strategy We performed paratuberculosis epidemiopathogenic phenotypic and genotypic characterization involving 24 SNPs in six candidate genes (*NOD2, CD209, SLC11A1, SP110, TLR2* and *TLR4*) on 502 slaughtered Friesian cows. In the current study, we investigate whether recently proposed paratuberculosis (PTB) epidemiopathogenic (EP) forms (apparently free-AF, latent-LAT and patent-PAT) could be associated with some combination of these 24 SNPs. Best EP form grouping was obtained using a combination of 5 SNPs in four genes (*CD209*: rs210748127; *SLC11A1*: rs110090506; *SP110*: rs136859213 and rs110480812; and *TLR2*: rs41830058). These groups were defined according to the level of infection progression risk to patent epidemiopathogenic forms and showed the following distributions: LOWIN (low) with 39 (8%) cases (94.9% AF/5.1% LAT/0% PAT); LATIN (low) with 17 (3%) cases (5.9% AF/94.1% LAT/0% PAT); AVERIN (average) with 413 (82%) cases (52.1% AF/38.5% LAT/9.4% PAT) and PATIN (patent) with 33 (7%) cases (36.4% AF/24.2% LAT/39.4% PAT). Age of slaughter was significantly higher for LATIN (88.3 months) compared to AVERIN (65.3 months; p = 0.0007) and PATIN (59.1 months; p = 0.0004), and for LOWIN (73.9 months) compared to PATIN (p = 0.0233), and nearly significant compared to AVERIN (p = 0.0572) These results suggest that some selected genetic polymorphisms have a potential use as markers of PTB EP forms and thus add a new tool for the control of this widespread infection.

## 1 Introduction

Chronic latent and patent *Mycobacterium avium* subsp. *paratuberculosis* (MAP) infections affecting domestic and wild ruminants represent a global major issue on animal health, recognized by the World Organization for Animal Health (OIE) (OIE, 2014) who requires member countries to maintain an epidemiological surveillance with notification of disease cases. This relevance has also been pointed out in a recent study where MAP has ranked among the top 100 domestic pathogens in Europe with an importance comparable to the Scrapie agent, according to their respective H-index scores (McIntyre et al., 2014).

Indeed, numerous sources consider MAP infections as highly prevalent between dairy cattle herds (USDA-APHIS-VS-CEAH, 2008; Nielsen and Toft, 2009; Frössling et al., 2013; Vazquez et al., 2014a). If the infection progresses to clinical disease, something often seen by farmers, it leads to a progressive impaired cow health status manifested mainly by weight loss and diarrhea, as well as by decreased milk production (Ott et al., 1999; Juste et al., 2009) and reduced life-span (Groenendaal et al., 2002; Alonso-Hearn et al., 2012), representing the most costly infectious disease affecting cattle (Singh et al., 2013).

Given all the above, it is widely advised to establish control measures against MAP in order to minimize its effects. Currently, the main disease-control strategy within dairy herds involves the combination of appropriate hygienic-sanitary measures and test and cull programs, (Groenendaal et al., 2002; Windsor and Whittington, 2010; Mortier et al., 2013; Garry, 2011). Since test and cull programs have been proven to be time-consuming, expensive, and eventually not as efficient as expected in part due to the lack of sensitivity of diagnostic tests (Nielsen and Toft, 2008), new approaches are needed. In this context, genetic Marker-Assisted Selection (MAS) and vaccination epigenetic increase of the individual resistance to paratuberculosis are highly promising tools. At present, the latter has achieved successful therapeutic and epidemiological outcomes (Bastida and Juste, 2011) by reducing MAP isolation in feces and tissues of infected animals and by increasing both milk production (Juste et al., 2009) and cow productive lifespan in infected farms (Alonso-Hearn et al., 2012), at highly efficient cost-effective rates compared to other strategies (Juste and Casal, 1993; Groenendaal and Galligan, 2003). However, given that interference with *Mycobacterium bovis* detection has been viewed up to now as an unacceptable effect in eradication of tuberculosis, most bovine tuberculosis eradication programs heavily restrict its use. Although those paratuberculosis (PTB) vaccine cross-reactions could be easily differentiated in most cases by use of the EU-official comparative intradermal test (CIT) (Garrido et al., 2013; Serrano et al., 2017), PTB vaccination use remains forbidden in most European countries, with the exception of the UK in specific cases and France from February 2014 [http://www.ircp.anmv.anses.fr/rcp.aspx?NomMedicament=SILIRUM. Accessed: 2016/11/24].

Genetic Marker-Assisted Selection (MAS) has been explored after steady advances in identifying genetic effects on resistance to bovine PTB, by both specific candidate gene (Pinedo et al., 2009a,b,c: Ruiz-Larrañaga et al., 2010a,b,c, 2011, 2012; Verschoor et al., 2010; Pant et al., 2011; Purdie et al., 2011; Küpper et al., 2014; Vazquez et al., 2014b; Sharma et al., 2015) and genome-wide association studies (GWAS) (Kirkpatrick et al., 2011; Purdie et al., 2011; Minozzi et al., 2012; van Hulzen et al., 2012; Alpay et al., 2014; Zare et al., 2014), especially over the last decade. Some of these candidate genes associated with MAP infections play a role in host innate immunity responses to MAP by encoding membrane-bound and cytoplasmic pattern recognition receptors (PRR) in macrophages, dendritic cells and other immune cells which enable the identification of mycobacterial pathogen-associated molecular patterns (PAMPs) such as *Nucleotide Oligomerization Domain 2* (*NOD2*) (Ruiz-Larrañaga et al., 2010a), *Dendritic Cell-Specific ICAM-3-Grabbing Nonintegrin Dectin-1* (*DC-SIGN* or *CD209*) (Ruiz-Larrañaga et al., 2012; Vazquez et al., 2014b), *Toll-like receptor (TLR)* (Ruiz-Larrañaga et al., 2011) genes, or by encoding molecules that modulate innate immune response against MAP as the *Solute Carrier Family 11 Member 1* (*SLC11A1*) (also known as *Natural Resistance-Associated Macrophage Protein 1*, or *NRAMP1*) gene (Ruiz-Larrañaga et al., 2010b). Moreover, the mouse *SP110* gene (*Ipr*) has been proposed to control mycobacterial replication in macrophages (Pan et al., 2005, Wu et al., 2015) and the bovine ortholog has already been associated with susceptibility to MAP infection (Ruiz-Larrañaga et al., 2010c).

Even so, it seems more plausible that genetic predisposition could be strongly determined by the interaction of more than one candidate gene, and even more after the recent evidence of epistasis among bovine innate immunity genes affecting susceptibility to MAP infection (Ruiz-Larrañaga et al., 2017). However, no combination of genetic markers that could explain individual differences in the risk of becoming infected has been suggested for this disease to date. In this sense, a critical step is the definition of the relevant forms of infection or disease (Osterstock et al., 2010; Küpper et al., 2014). Although classifications of PTB in small ruminants and cattle have been around since mid XXth century (Stamp and Watt, 1954; Buergelt et al., 1978; Paliwal and Rajya, 1982; Clarke and Little, 1996; Pérez et al., 1996; Corpa et al., 2000; González et al., 2005), none of them has been used to successfully search for a useful genetic combination.

In the current study we propose to follow a similar approach to that accepted for Scrapie infections in small ruminants, in which different Scrapie risk groups are defined according to their prion protein (PrP) genotype for codons 136, 154 and 174 (Dawson et al., 1998), in order to identify polygenic effects on resistance and susceptibility to PTB. This, coupled with the use of a recently proposed pathological classification grounded on the hypothesis that innate immunity is usually reflected by inflammatory manifestations in target organs better identified by histopathological changes (Vazquez et al., 2014a), led us to search for an association between different combinations of selected SNPs in innate immunity-associated genes and these newly defined paratuberculosis epidemiopathogenic forms, that could work as a new approach for paratuberculosis control by coupling it to cattle breeding programs.

## 2 Material and methods

### 2.1 Animals and genotype data

Genotype data of the 502 Holstein-Friesian animals used in the present study belong to a previous larger study (Vazquez et al., 2014b). Animals were firstly phenotyped for MAP infections using immunological, microbiological and histopathological techniques and classified into three PTB status (apparently free of infection, latent PTB and patent PTB) according to Vazquez et al. (2014a). In total, 265 controls (apparently free) and 237 cases (78.1% latent PTB and 21.9% patent PTB) were considered in this study.

A more detailed description of animals (age and origin), sampling method, compliance with legislation on animal welfare, permission to collect and use the samples has been published elsewhere (Vazquez et al., 2012, 2013, 2014a,b). Age at slaughter, data collection and methodology for PTB ELISA, interferon-gamma (IFN-g) release assay (IGRA), and MAP isolation were performed as described elsewhere (Vazquez et al., 2014a)

Genomic DNA was typed for 24 SNPs in six innate immune related genes previously associated with paratuberculosis (Ruiz-Larrañaga et al., 2010a,b,c, 2011, 2012, Vazquez et al., 2014b): *CD209* (rs208222804, rs209491136, rs211654540, rs208814257, and rs210748127), *NOD2* (rs109601360, rs43710288, rs43710289, and rs43710290), *SLC11A1* (rs109453173, rs110090506), *SP110* (rs136859213, rs133080973, and rs110480812), *TLR2* (rs110491977, rs68268259, rs41830060, rs109971269, rs41830058, rs43706434, and rs43706433), and *TLR4* (rs29017188, rs43578097, and rs43578100). The SNPs were genotyped using the TaqMan® OpenArray® technology (Life Technologies; Carlsbad, USA) according to the manufacturer's indications, and Autocaller v1.1 software (Life Technologies; Carlsbad, USA) for allele assignation. All individuals and SNP fit the three quality control parameters used elsewhere (Vazquez et al., 2014b): sample call rate (≥80%), SNP call rate (≥80%), and Hardy-Weinberg equilibrium (HWE).

### 2.2 Genetic risk classification for latent and patent PTB

**Fig. 1:**
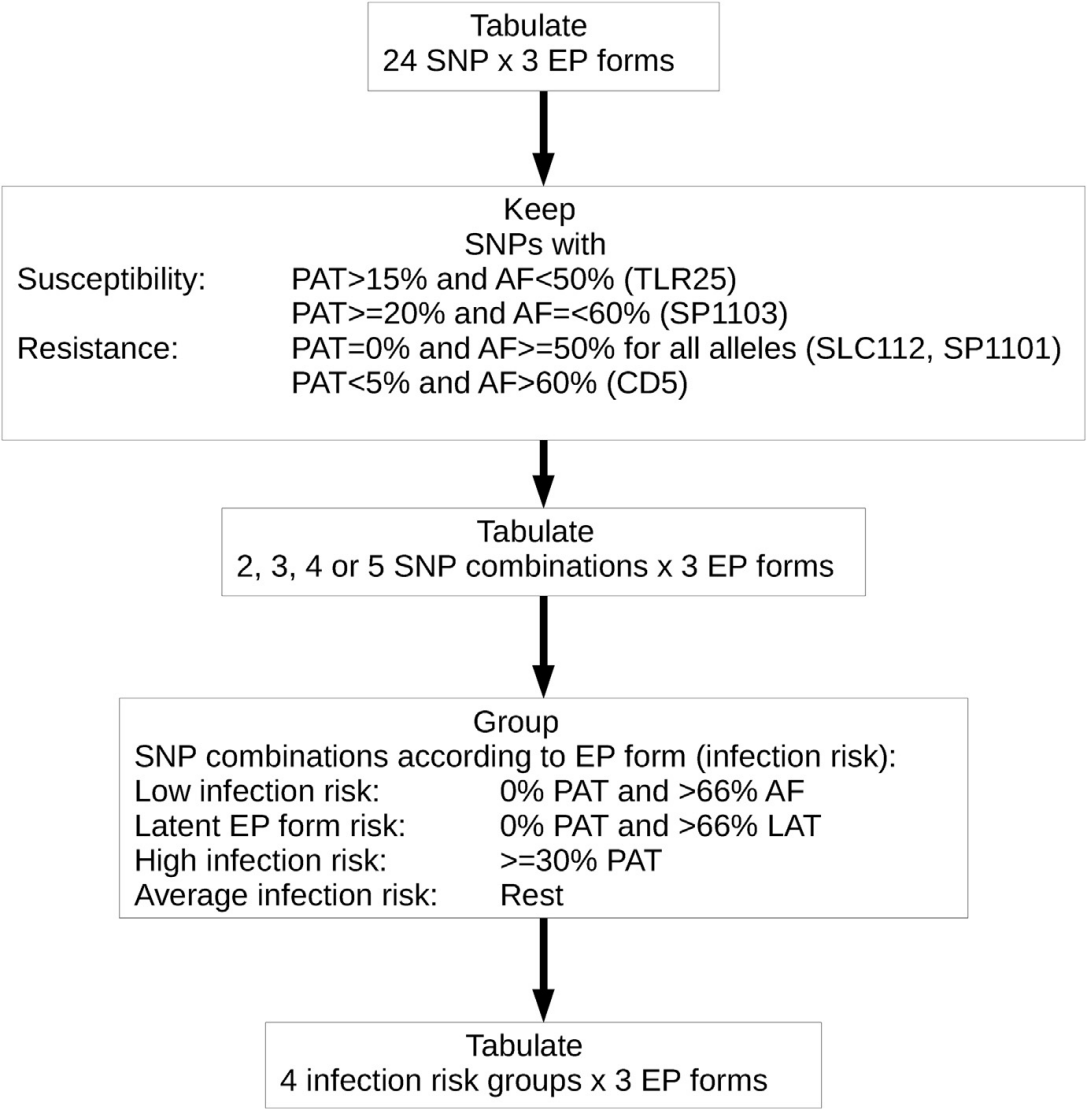
Flow chart for risk group generation. PAT: Patent epidemiopathogenic (EP) form; AF: Apparently free EP form; LAT: Latent EP form; LOWIN: Apparently free EP form risk or low infection progression risk; LATIN: Latent EP form risk or latent infection progression risk; PATIN: Patent EP form risk or infection progression high risk; AVERIN: SNP combinations that did not fit into any of the other risk groups.

The procedure of SNP selection and combination is depicted in Fig. 1. First, SNPs were individually filtered according their association with the two ends of the disease resistance-susceptibility range. The resistance end included SNPs with a frequency of apparently free (AF) forms equal or greater than 50% and no patent (PAT) forms for all alleles as well as those with over 60% AF forms and less than 5% PAT forms for, at least, one allele. The susceptibility end consisted of SNPs with more than 15% PAT forms and less than 50% AF forms or with 20% or more PAT forms and 60% or less AF forms. This filter was established as one yielding a number of SNP that was manageable for the next step. This next step consisted in building frequency distributions for different SNP combinations (from 2 to 5) with a similar EP form frequency distribution. According to this, SNP combinations were grouped if they had: a) no PAT form and more than 66% AF forms (low infection risk); b) no PAT form and more than 66% LAT form (latent EP form risk); c) 30% or more PAT forms (high infection risk). Only SNP combinations of 5 complied with these conditions and were subsequently grouped in the following categories and frequencies:

- Apparently free EP form risk or low infection progression risk (LOWIN): Over 90% of individuals with this genetic profile were apparently free of infection.
- Latent EP form risk or latent infection progression risk (LATIN): Over 90% of individuals with this profile showed latent forms of PTB.
- Patent EP form risk or infection progression high risk (PATIN): Patent forms in this group accounted for about 40% of its individuals.

SNP combinations that did not fit into any of the above mentioned groups assigned to a fourth group considered of an average risk of infection and named AVERIN.

### 2.3 Statistical analyses

To test whether the genetic risk groups were associated to other variables expected to be modified by PTB, an analysis of variance was run for ELISA optical density (OD) readings and positivity rate, IFN-g OD, MAP isolation frequency and age at slaughter with the GLM procedure of the SAS 9.1 statistical package. Then the risk level means were compared to the control (AVERIN) or the PATIN risk group with the Dunnett test. Statistical analyses resulting in p-values lower than 0.05 were considered to be significant.

## 3 Results

A total of five SNPs in *rs210748127 (CD209), rs110090506 (SLC11A1), rs41830058 (TLR2), rs136859213 and rs110480812 (SP110)* bovine genes were found to comply with the defined criteria. As result, 502 animals without any missing genotype for those 5 SNP were considered for subsequent analyses.

A total of 70 genotype combinations were built for those SNP and grouped based on the four established PTB risk levels, as shown in Table 1. Twelve genotype combinations were included in the LATIN group, 7 in the PATIN, 24 in the LOWIN, and 27 in the AVERIN.

**Table 1:** Progression risk groups genotype and individual frequencies

	Genotype combinations		Cows	
	N	%	N	%
LOWIN	24	34.3	39	7.77
LATIN	12	17.1	17	3.39
AVERIN	26	38.6	416	82.87
PATIN	8	10.0	30	5.98
Total	70	100	502	100

LOWIN: Apparently free EP form risk or low infection progression risk. LATIN: Latent EP form risk or latent infection progression risk. PATIN: Patent EP form risk or infection progression high risk. AVERIN: SNP combinations that did not fit into any of the above mentioned groups.

The LOWIN group of 39 animals was finally constituted (94.9%) by animals epidemio-pathologically defined as AF. The 17 cows included in the LATIN were 94.1% LAT forms. The PATIN was the group that had the highest rate of PAT forms, which finally accounted to 40.0% (n = 12/30). The remaining genotypes for which no higher proportions of any PTB EP form could be obtained, were defined as of average risk (AVERIN) and contained the vast majority of cases in the study (n = 416) (Table 2). About 10% of individuals in this AVERIN category show patent (PAT) forms of PTB. This prevalence was found to be the average prevalence in the most recent large slaughterhouse study carried out in the Basque Country (Vazquez et al., 2014a) and fits with other estimates (Garrido, 2001).

**Table 2:** Frequency of epidemiopathogenic (EP) forms (apparently free-AF, latent-LAT and patent-PAT) according to progression risk level.

	AF		LAT		AF + LAT	PAT	
	N	%	N	%	N/%	N	%
LOWIN	**37**	**94.9**	**2**	**5.1**	39/100%	**0**	**0.0**
LATIN	**1**	**5.9**	**16**	**94.1**	17/100%	**0**	**0.0**
PATIN	**10**	**33.3**	**8**	**26.7**	18/60%	**12**	**40.0**
AVERIN	**217**	**52.2**	**159**	**38.2**	376/90.4%	**40**	**9.6**
Total	**265**	***52.8***	**185**	***36.9***	450/89.6%	**52**	***10.4***

LOWIN: Apparently free EP form risk or low infection progression risk. LATIN: Latent EP form risk or latent infection progression risk. PATIN: Patent EP form risk or infection progression high risk. AVERIN: SNP combinations that did not fit into any of the above mentioned groups. Bold values indicates percentages.

**Fig. 2:**
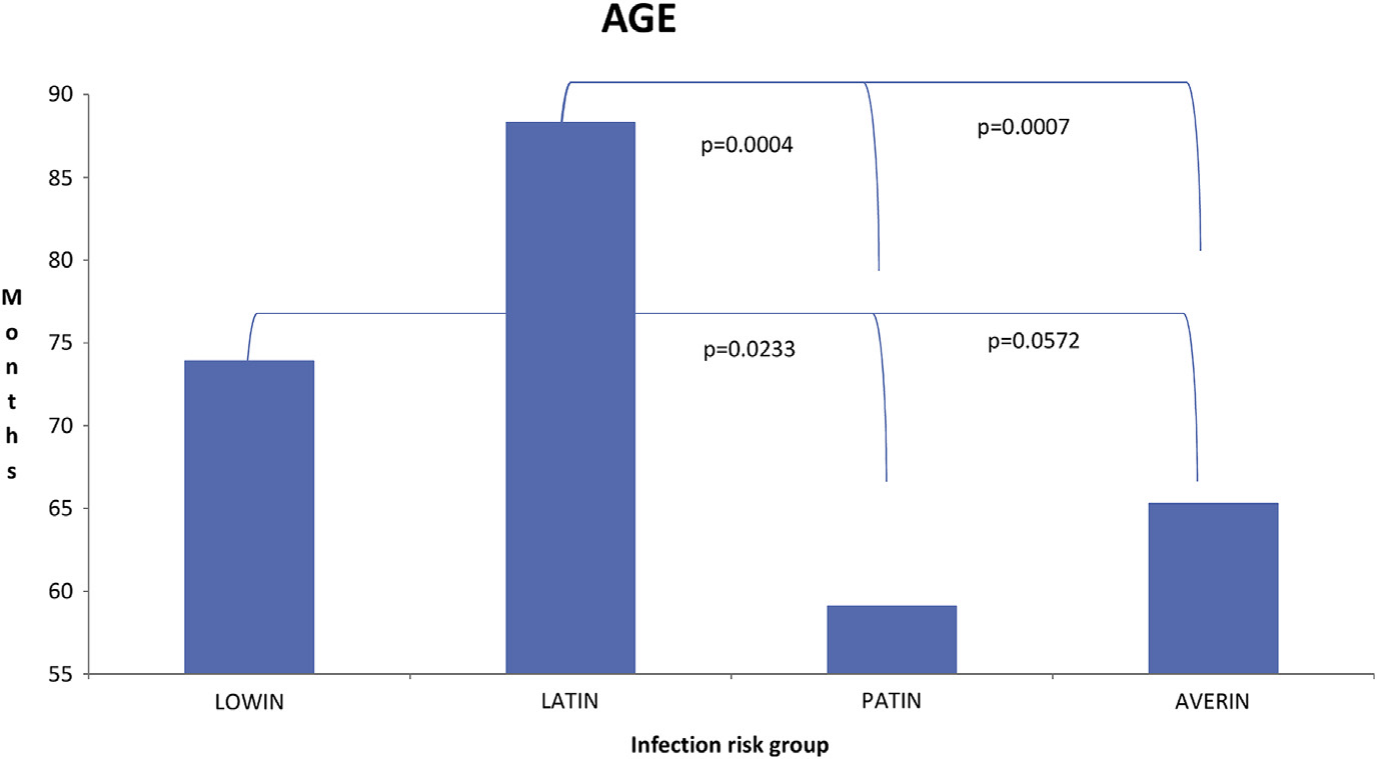
Effect of genetic risk of paratuberculosis infection progression on age at slaughter (mean months ± standard error). (LOWIN = 73.92 ± 4.32; LATIN = 88.30 ± 6.60; PATIN = 59.10 ± 5.11; AVERIN = 65.29 ± 1.34). Note the most significant increments on life-span for the LATIN group compared to AVERIN (35%) and PATIN (49%) groups. LOWIN: Apparently free EP form risk or low infection progression risk; LATIN: Latent EP form risk or latent infection progression risk; PATIN: Patent EP form risk or infection progression high risk; AVERIN: SNP combinations that did not fit into any of the other risk groups.

Age of slaughter was significantly higher for LATIN (88.3 months) compared to AVERIN (65.3 months; p = 0.0007) and PATIN (59.1 months; p = 0.0004), and for LOWIN (73.9 months) compared to PATIN (p = 0.0233), and nearly significant compared to AVERIN (p = 0.0572) (Fig. 2).

**Fig. 3:**
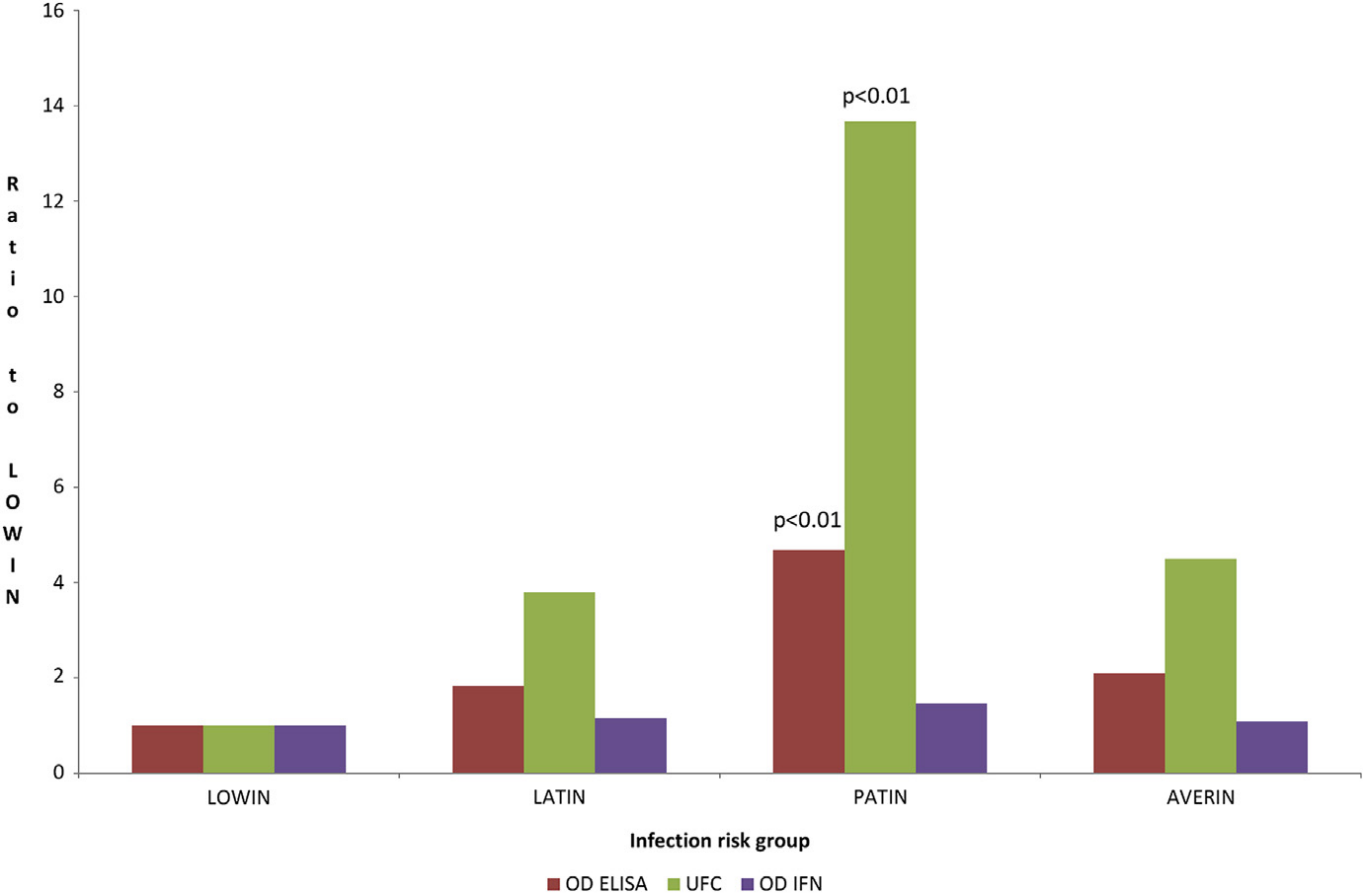
Paratuberculosis specific immunological and microbiological variables according to progression risk. OD ELISA= Optical density in paratuberculosis specific ELISA. UFC = colony forming units in tissue culture. OD IFN = Optical density in the MAP-specific interferon gamma release assay. Note the increased OD values for anti-MAP specific antibodies detection by ELISA and MAP colonies isolation between PATIN and LOWIN infection risk groups. LOWIN: Apparently free EP form risk or low infection progression risk; LATIN: Latent EP form risk or latent infection progression risk; PATIN: Patent EP form risk or infection progression high risk; AVERIN: SNP combinations that did not fit into any of the other risk groups.

Fig. 3 summarizes PTB specific immunological and microbiological variables according to progression risk. Notably, OD ELISA values and MAP load were higher for PATIN when compared to those for LOWIN infection risk group (p < 0.01).

## 4 Discussion

Our results seem to match what can be expected from a genetically controlled character in a population. Even simplifying into resistant and susceptible, our results show a resistant tail of about 11% (LOWIN and LATIN), and another one of susceptible animals of about 6% animals with the PATIN genotype. The remaining 83% would be a mix of animals with different degrees of resistance or susceptibility. They show a noticeable association between a series of Friesian cattle genotype combinations in several selected innate immunity genes and EP forms of PTB (Tables 1 and 2). These findings, although preliminary, are relevant, first because they are consistent with the hypothesis that PTB as other mycobacteriosis and intracellular infections are modulated by genetically regulated innate immunity mechanisms, and second, because they are a proof of concept that a combination of genetic features can help to select the more resistant animals and to exclude from breeding those that are most likely to become advanced forms and clinical cases of PTB. In fact, a recent study has already suggested that gene-gene interaction may be conditioning susceptibility to MAP infection in the bovine species (Ruiz-Larrañaga et al., 2017).

This study is the first one to show such a definite association and we think that the main reason is the way in which the phenotypes are defined. That is, instead of looking into indirect immune variables or microbiological indicators that might be biased by the widespread presence of MAP in the cattle environments in which the bovine species is bred, we have made an attempt to look into the more direct expression of the innate immunity mechanisms of mycobacterial pathogenesis which is tissue chronic inflammation in agreement with strong recommendations for adequate definitions of disease phenotypes that are found in the literature (Osterstock et al., 2010; Küpper et al., 2014). Even though the number of studied SNPs is limited, the associations are not all-in/all-out and not based in a large sample of cases, they seem to be enough to point out a new way to build a hypothesis worth of testing in a wider population.

From an epidemiological view, latent PTB represent a constant and unnoticed threat while patent PTB is a less prevalent form of infection, but with more adverse effects on body condition and productivity rates (milk production, carcass weight and age at culling) of infected animals, as well as on environmental mycobacterial loads (Vazquez et al., 2012, 2014a). Despite the complexity of immunopathogenetic mechanisms occurring in both types of MAP infections, in this paper a novel polygenic risk-classification for bovine PTB has been devised in order to provide dairy breeders promising guides to rear animals that are likely to have a lower disease incidence within the herd.

To protect dairy cattle from MAP infections has been an arduous challenge to deal with that nowadays still needs to be efficiently addressed. The combination of hygienic-sanitary measures and test and culling has shown its limits (Garry, 2011), and new approaches are needed in order to increase the efficiency of PTB control programs and to make them less cost and time-consuming. Since pathogen, host and environmental factors are decisive for determining the risk of MAP infections, all these variables should be considered. SNPs genotyping can be precisely used as a tool to better evaluate host-pathogen immunopathological interactions for control purposes. Our results suggested that *CD209, SLC11A1*, *SP110* and *TLR2* genes play a pivotal role in determining risk to MAP infection epidemiopathogenic forms in Spanish Holstein-Friesian cattle. The identification of these four genes as risk markers of bovine PTB confirms previous findings in candidate gene studies (Ruiz-Larrañaga et al., 2010b,c, 2011, 2012, 2017; Vazquez et al. 2014b). However, it is remarkable that even though a *NOD*2 polymorphism showed up as related to epidemiopathogenic forms, adding it to the five SNPs selected in this study did not improve the distribution (data not shown) in spite of previous associations between this gene and MAP shedding for this breed (Ruiz-Larrañaga et al., 2010a; Küpper et al., 2014) and its more similar disease in humans, Crohn's disease (Hugot et al., 2001; Ogura et al., 2001).

These results may be applicable to other Holstein-Friesian populations from other dairy producing countries because the general trend through genetic selection has been mainly focused on increasing milk production, which has contributed to worldwide homogenize the genetic profile of this breed thanks to extensive international trade of frozen semen. This would, in turn, also give an outline of the scope of possible applications of this novel classification and its global benefit effects by promoting more resistant dairy cattle populations worldwide.

According to our findings, knowing the genetic profile for the five proposed SNPs could help decision making on selecting calves, heifers, cows and bulls for breeding or on the purchase of new animals. Hence, SNP genotyping would be required just once, which would not result into too expensive costs, and would allow to establish different models of breeding, from the stricter ones allowing only those most resistant genotypes to others more flexible just considering the exclusion of more susceptible genotypes, depending on both the aims and resources of each farm.

Although knowing the genetic predisposition will not eradicate MAP infections *per se* because of their environmental component, among other factors, it could be expected to reduce the individual prevalence of PTB as well as to increase the level of genetic resistance in dairy cattle population by promoting the breeding of those more resistant genotypes, and thereby reducing MAP infection pressure in some way, similarly to the experiences learnt from Scrapie breeding and control programs (Hagenaars et al., 2010; Nodelijk et al., 2011). Indeed, from a public health perspective, the fact that human exposure to MAP could be in the long run be reduced as a consequence of this selection model could be understood as an adequate cautionary measure by the general population who is increasingly concerned about the potential zoonotic role of this mycobacterial agent in the pathogenesis of Crohn disease (Naser et al., 2014) and the presence of MAP in food products (Groenendaal and Zagmutt, 2008).

Furthermore, taking into account that innate immune response act as a first barrier to pathogens, it will remain to be evaluated whether any beneficial effect on preventing other infections, both specific, such as *Mycobacterium bovis* or unspecific, like mastitis, could be also provided by breeding for the lower risks combinations of these candidate SNPs.

In conclusion, although the work reported here has substantial limitations in the number of SNPs and the sample size, that need to be validated in a more specific study, our results open the way to considering an specific combination of SNPs (C*D209*, *SLC11A1*, *TLR2*, *SP1101* and *SP1102*) in dairy cattle breeding selection programs as an effective control strategy against paratuberculosis at an affordable cost.

## Declarations

### Author contribution statement

Ramon Juste: Conceived and designed the experiments; Analyzed and interpreted the data; Wrote the paper.

Joseba Garrido: Conceived and designed the experiments; Performed the experiments.

Andone Estonba: Conceived and designed the experiments.

Patricia Vazquez: Performed the experiments; Analyzed and interpreted the data; Wrote the paper.

Otsanda Ruiz-Larrañaga, Elena Molina, Maria Adoracion Cortés Alonso, Nieves Gomez: Performed the experiments.

Marivi Geijo: Performed the experiments; Contributed reagents, materials, analysis tools or data.

Marta Alonso-Hearn: Analyzed and interpreted the data; Wrote the paper.

Mikel Iriondo, Mikel Agirre, Carmen Manzano, Iker Sevilla, Valentin Perez: Contributed reagents, materials, analysis tools or data.

### Funding statement

This work was supported by the Ministry of Economy and Competitiveness (MINECO) (projects AGL2006-14315-C02 and RTA2014-00009), Basque Government (GV/EJ) (SAIOTEK program: SA-2010/00102), European Regional Development Fund (ERDF), and European Social Fund (ESF) is also gratefully acknowledged. Patricia Vázquez was holder of a graduate fellowship award (FPI) (BES-2007-17170) from the Spanish MINECO.

### Competing interest statement

The authors declare no conflict of interest.

### Additional information

No additional information is available for this paper.
